# Demographic Factors Influencing the Need for Repeat Radiofrequency Ablation in Chronic Pain Management: A Retrospective Study

**DOI:** 10.7759/cureus.116278

**Published:** 2026-09-15

**Authors:** John Abdallah, Navdeep S Manhas, Shadman Hyder, Mohammad M Ali, Lauren Truong, Nikoli Brown, Krish T Bharat, Duc A Tran, Eugene Pak

**Affiliations:** 1 Physical Medicine and Rehabilitation, California University of Science and Medicine, Colton, USA; 2 Physical Medicine and Rehabilitation, Loma Linda University Medical Center, Loma Linda, USA; 3 Pain Medicine, University of Texas, San Antonio, San Antonio, USA; 4 Physical Medicine and Rehabilitation, University of Pittsburgh Medical Center, Pittsburgh, USA; 5 Pain Medicine, Loma Linda University Medical Center, Loma Linda, USA

**Keywords:** chronic pain, interventional pain, pain management, radiofrequency ablation, retrospective cohort

## Abstract

Background: Radiofrequency ablation (RFA) is a common noninvasive intervention for chronic pain management; however, the large variability in patient response underscores the need to better understand factors impacting treatment and patient outcomes. This study investigated whether demographic variables, specifically race, sex, age, and body mass index (BMI), are associated with the frequency of repeat RFA procedures in patients with chronic pain.

Methods: This retrospective case series study was conducted at a single outpatient academic pain management clinic. A total of 170 clinic patients (mean age: 58.8 years; mean BMI: 31.5) who underwent at least two radiofrequency ablation (RFA) procedures between January 2019 and January 2023 were included. All patients had complete demographic and procedural data and at least one follow-up visit after their initial RFA; patients with incomplete or missing records or no follow-up visits were excluded. The primary outcome measure was the number of RFA procedures per patient.

Results: One-way analysis of variance (ANOVA) demonstrated no statistically significant differences in the number of repeat RFA procedures across racial groups (F(4,165) = 0.58, p = 0.68) or between genders (F(1,168) = 1.85, p = 0.18). Linear regression analysis found no statistically significant associations between number of repeat RFA procedures and age (F(1,168) = 3.06, p = 0.08) or BMI (F(1,166) = 0.00, p = 0.95). Ordered logistic regression further illustrated that the demographic variables examined - race, sex, age, BMI - were not associated with the number of repeat RFA procedures (χ²(4) = 5.53, p = 0.24).

Conclusions: Overall, our results showed that demographics including sex, race, age, and BMI were not associated with the frequency of RFA procedures in chronic pain management. Other variables like procedural variance, social support, or other comorbidities may serve as better potential determinants for treatment response and pain outcomes. These findings suggest that demographic variables alone may not explain variability in repeat RFA utilization.

## Introduction

Chronic pain, defined as pain that persists or recurs for longer than three months, is a disabling condition that substantially limits patients’ daily functioning and quality of life [1]. In 2021, an estimated 51.6 million U.S. adults (20.9% of the population) experienced chronic pain, with approximately 17.1 million adults reporting high-impact chronic pain [2]. Furthermore, chronic pain has been associated with increased rates of dementia, depression, substance use disorders, and suicide, underscoring the urgent need for effective long-term management strategies [2,3]. Owing to the multifactorial nature of chronic pain, treatment is often complex and requires an individualized, multidisciplinary biopsychosocial approach [4]. As the limitations of pharmacologic therapies-including dependence, tolerance, overdose risk, and systemic adverse effects-have become increasingly recognized, attention has shifted toward minimally invasive interventional procedures such as radiofrequency ablation (RFA), which may provide durable pain relief with fewer systemic side effects [5,6].

RFA is a minimally invasive interventional procedure that provides targeted relief for musculoskeletal and neuropathic pain through interruption or modulation of nociceptive pathways [7]. The procedure involves generating thermal lesions using alternating electrical current, typically at temperatures of 80-90°C, delivered through an ablation electrode to induce coagulative necrosis and axonal destruction, thereby interrupting nociceptive signal transmission [8]. In addition to conventional RFA, modified techniques such as cooled RFA and pulsed RFA have emerged as effective treatment modalities for neuropathic pain conditions [9,10]. Cooled RFA and pulsed RFA have demonstrated favorable outcomes in clinical scenarios requiring larger lesion coverage and neuromodulation, respectively [8,11]. These procedural variations allow treatment strategies to be tailored according to the targeted nerve distribution and underlying pain mechanism.

RFA is indicated for patients with chronic pain refractory to conservative management, including exercise, physical therapy, and pharmacologic interventions, particularly when pain can be localized to a specific sensory nerve target [8,12]. Notably, RFA has demonstrated pain relief lasting six to 12 months across multiple chronic pain conditions, with some studies suggesting sustained benefits for up to 24 months in certain conditions [6]. Compared with opioid-based therapies and more invasive surgical interventions, RFA may provide durable analgesia with fewer adverse effects and lower procedural risk [5,6,8]. Established indications for conventional thermal RFA include facet-mediated low back pain, confirmed by positive diagnostic medial branch blocks [8,12]. Furthermore, pulsed RFA has demonstrated broader utility, with applications in radicular pain, discogenic pain, neuralgia, and post-amputation pain syndromes involving peripheral nerves such as the suprascapular, intercostal, and pudendal nerves [8]. More recently, RFA has also demonstrated promising outcomes in the treatment of knee osteoarthritis and other chronic pain syndromes [8,9,13].

Despite these favorable outcomes, patient response to RFA remains variable, with some individuals requiring repeat procedures because of nerve regeneration, symptom recurrence, or incomplete lesioning of the targeted nerve [6]. In addition, psychiatric comorbidities and demographic factors such as sex and obesity may influence treatment response and procedural durability [12,14]. This variability in clinical outcomes highlights the need for further investigation into factors associated with repeat RFA utilization. 

The objective of this study was to investigate whether demographic variables, including race, sex, age, and body mass index (BMI), were associated with the need for repeat RFA procedures in patients with chronic pain. Improved understanding of factors associated with repeat RFA utilization may help guide individualized treatment planning and optimize long-term pain management strategies.

## Materials and methods

This retrospective study was conducted at Loma Linda University, looking at its Pain Management Clinic’s patient data from January 2016 to December 2023. Data were collected exclusively from this single site and did not include other affiliated or satellite clinics. Approval was obtained to proceed through the Loma Linda University Institutional Review Board (IRB) with Human Research and Compliance and was given the designation IRB# 5250634. Patient data was de-identified to ensure confidentiality and limit biases. Due to the retrospective nature of the study, informed consent was not required.

Inclusion criteria for the study included patients older than 18 years, patients who had received their initial RFA within the designated time range, patients with documented demographic data (race, age, sex, and body mass index), and patients who were diagnosed with either facetogenic low back disease or knee osteoarthritis prior to the procedure. Exclusion criteria included protocol violations such as missing or failed prerequisite diagnostic blocks, unknown initial RFA treatment dates, and care outside the given time range. Prerequisite diagnostic confirmation consisted of one or two diagnostic medial branch blocks for facetogenic pain or genicular nerve blocks for knee osteoarthritis, performed per clinic protocol prior to RFA. Failed diagnostic blocks were defined as inadequate pain relief as documented in the medical record. Power analysis was completed a priori to calculate a required sample size to achieve a power level of 95%. We based such calculations on an alpha value of 0.05, an anticipated effect size of 0.15, and four predictor variables. After inputting the above data into the statistical program, we determined that a sample size of at least 129 participants is required to achieve the aforementioned power level.

Out of the 171 screened patients, 170 patients were included in the study, with one patient excluded based on the study’s exclusion criteria. Data collection was performed by two resident physicians and a medical student, with oversight by a supervising attending physician to standardize the screening process. Due to the retrospective design of the study, data collectors were not blinded to the study hypothesis. Formal inter-rater reliability analysis was not performed. Any discrepancies in data abstraction were resolved through discussion and consensus among the investigators and supervising attending physician.

Data were recorded in Google Sheets (Google LLC, Mountain View, CA, USA) and were analyzed using STATA version 3 (StataCorp LLC, College Station, TX) by a statistician who was provided coded data. Descriptive statistics (ranges, means, percentages) were used to summarize key patient characteristics. ANOVA was used to look at the number of RFAs (a count outcome) by race, along with the number of RFAs by sex (both sex and race being categorical variables with multiple groups). Linear regression was used to look at the number of RFAs compared to both age and BMI (both continuous). Finally, an ordinal logistic regression was also applied to look at all the demographic data.

To assess whether demographic factors were associated with the number of repeat RFA procedures, an ordered logistic regression model was performed. The number of repeat RFAs, the dependent variable, was categorized into ordinal groups based on increasing procedural frequency (e.g., 2 procedures, 3 procedures, ≥4 procedures) to evaluate whether demographic factors were associated with progressively greater RFA utilization. Ordered logistic regression was selected due to the ordered nature of these categories and the non-normal distribution of the count data. Independent variables included race, sex, age, and BMI. Race and sex were entered as categorical predictors, while age and BMI were treated as continuous variables. The model estimated the log-odds of undergoing a higher number of RFA procedures based on each demographic variable, controlling for the others. Statistical analyses were conducted using STATA version 3 (StataCorp LLC, College Station, TX) with significance set at p < 0.05. The proportional odds assumption was not formally assessed, which represents a limitation of the analysis.

## Results

Of the 170 included patients, 82 were male, and 88 were female. The age range of patients was from 18 years to 91 years, with a mean age of 58.8 years. The mean BMI of patients was 31.5. To categorize race, patients were allocated into distinct groups including Caucasian, Hispanic, African American, Asian, and other. The racial breakdown of patients was as follows: 74% Caucasian, 14% Hispanic, 10% African American, 1% Asian, and finally 1% were classified as other (Table 1).

**Table 1:** Demographic and clinical characteristics of the study cohort (N = 170)

Variable	n (%) or Mean ± SD	Range
Male	82 (48.2%)	—
Female	88 (51.8%)	—
Average age (years)	58.8	18–91
Average BMI (kg/m²)	31.5	—
Race		
Caucasian	126 (74.1%)	—
Hispanic	24 (14.1%)	—
African American	17 (10.0%)	—
Asian	2 (1.2%)	—
Other	1 (0.6%)	—

There was no significant difference in the number of repeat RFAs among racial groups, F(4,165) = 0.58, p = 0.676, η² = 0.014, indicating a small effect size. There was no significant difference in the number of repeat RFAs between sexes, F(1,168) = 1.85, p = 0.175, η² = 0.011, indicating a small effect size (Figure 1).

**Figure 1:**
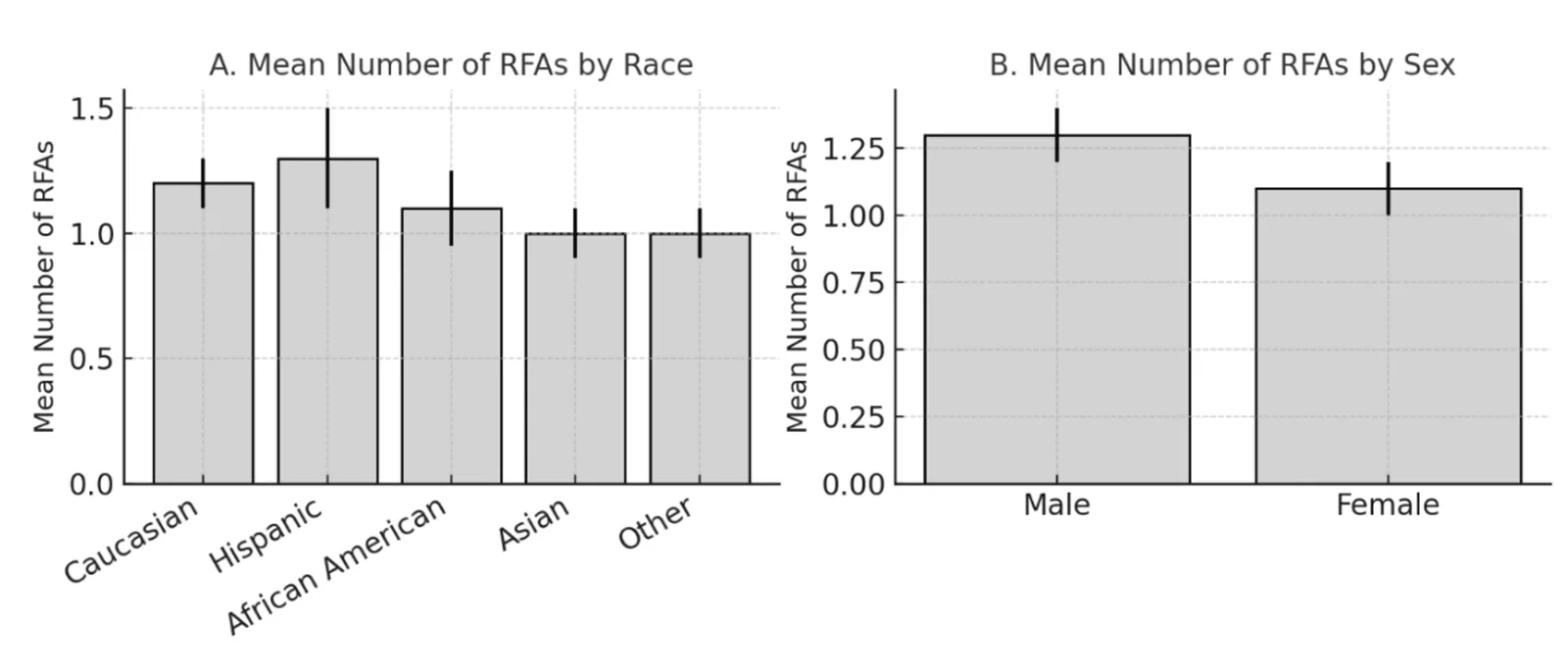
Bar charts showing mean number of repeat RFA procedures by race (panel A) and sex (panel B) RFA: radiofrequency ablation

Linear regression was performed to see whether age was associated with the number of RFA procedures. The overall regression model was not statistically significant, F(1,168) = 3.06, p = 0.082, 95% confidence interval (CI) (-0.046, 0.003) and explained only a small proportion of the variance in repeat RFA frequency (R² = 0.018). Linear regression was performed to see whether BMI was associated with the number of RFA procedures. The overall regression model was not statistically significant, F(1,166) = 0.00, p = 0.952, 95% CI (-0.049, 0.053) and explained none of the variance in repeat RFA frequency (R² = 0.000) (Table 2).

**Table 2:** Statistical analysis of demographic predictors of repeat RFA procedures RFA: radiofrequency ablation

Analysis	Predictor	Test statistic	df	p-value	95% CI	R²/Pseudo R²
ANOVA	Race	F = 0.58	-4,165	0.676	—	—
ANOVA	Sex	F = 1.85	-1,168	0.175	—	—
Linear regression	Age	F = 3.06	-1,168	0.082	(−0.046, 0.003)	0.018
Linear regression	BMI	F = 0.00	-1,166	0.952	(−0.049, 0.053)	0
Ordered logistic regression	Race, sex, age, BMI	χ²(4) = 5.53	—	0.237	—	0.0097

An ordered logistic regression analysis was conducted to examine whether demographic variables- race, sex, age, and body mass index (BMI)-were associated with the number of repeat radiofrequency ablation (RFA) procedures. The overall model was not statistically significant, χ²(4) = 5.53, p = 0.24, with a pseudo R² of 0.0097. None of the individual predictors showed significant associations with RFA frequency: race (β = −0.20, p = 0.33), sex (β = −0.46, p = 0.11), age (β = −0.013, p = 0.18), or BMI (β = −0.007, p = 0.70) (Figure 2).

**Figure 2:**
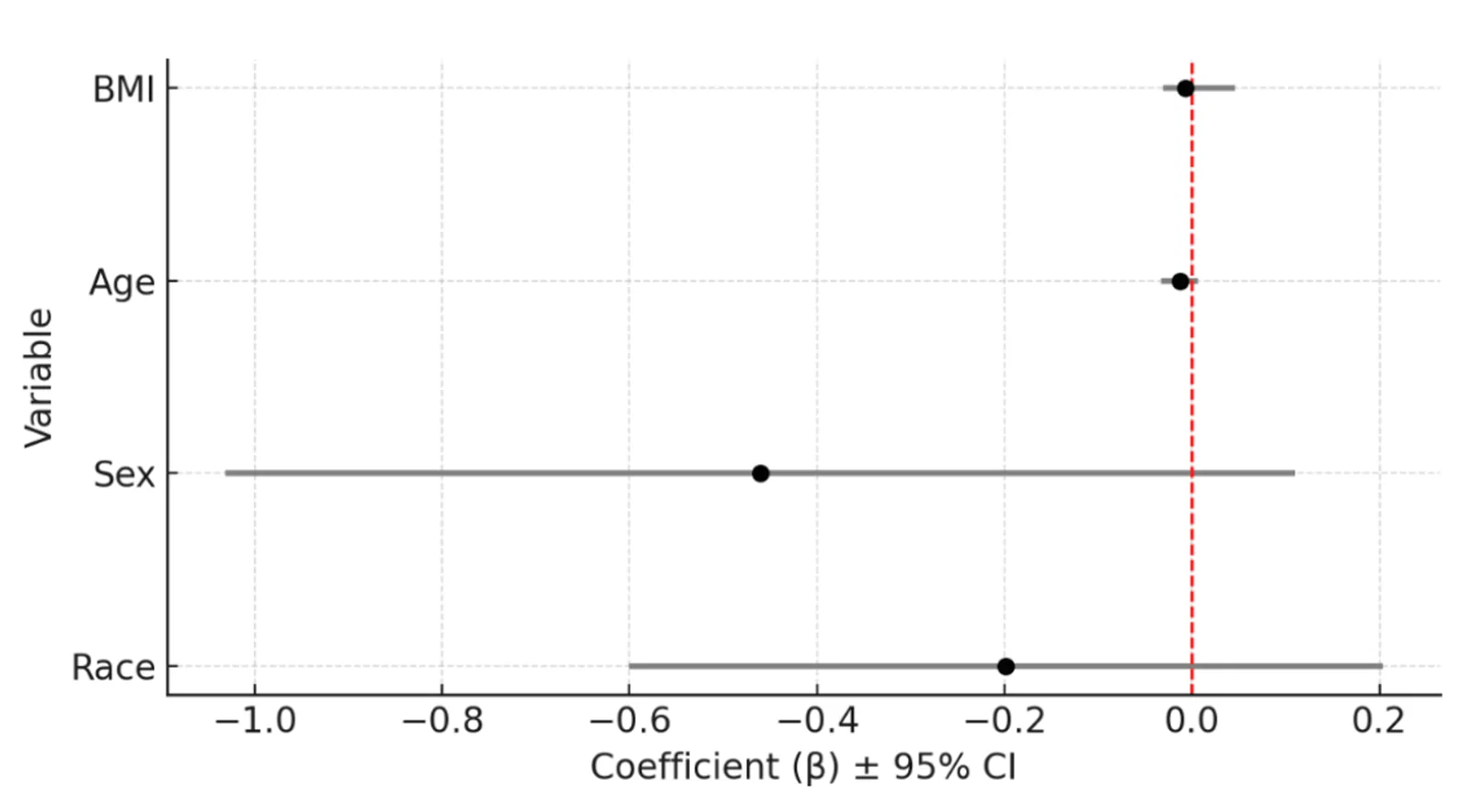
Ordered logistic regression coefficients for demographic predictors

These findings indicate that demographic characteristics were not significant predictors of the likelihood of undergoing multiple RFA procedures in this sample (Table 3).

**Table 3:** Ordered logistic regression examining demographic predictors of repeat RFA procedures RFA: radiofrequency ablation

Variable	Coefficient (β)	z	p-value	95% confidence interval
Race	−0.20	−0.97	0.33	−0.60 to 0.20
Sex	−0.46	−1.58	0.11	−1.03 to 0.11
Age	−0.013	−1.34	0.18	−0.033 to 0.006
BMI	−0.007	0.38	0.7	−0.031 to 0.046

## Discussion

This retrospective study evaluated whether demographic variables, including race, sex, age, and BMI, were associated with the frequency of repeat radiofrequency ablation (RFA) procedures in patients undergoing chronic pain management. Across multiple statistical models, including one-way ANOVA, linear regression, and ordered logistic regression, no statistically significant associations were identified between demographic characteristics and repeat RFA utilization. These findings suggest that demographic variables alone may have limited predictive value in determining the likelihood of requiring multiple RFA procedures in patients with chronic pain. RFA implementation for chronic musculoskeletal and neuropathic pain management provides a prolonged analgesic effect while minimizing the adverse systemic effects associated with pharmacologic therapies. Nevertheless, therapeutic response to RFA remains variable, and identifying factors associated with therapeutic durability remains clinically significant.

Prior investigations have explored whether demographic characteristics influence RFA outcomes, although findings have been inconsistent. Some studies have reported improved pain outcomes among female patients and individuals with lower BMI, whereas others have failed to demonstrate significant relationships between demographic variables and RFA efficacy or recurrence rates [14-19]. Our findings are more consistent with the latter body of literature and suggest that successful RFA intervention may be strongly influenced by other factors. The absence of significant associations between age and repeat RFA frequency is notable, given the physiologic and degenerative changes associated with aging that may exacerbate chronic pain severity. Similarly, the lack of association between BMI and repeat procedural frequency contrasts with prior studies suggesting obesity may negatively affect procedural outcomes through weakened biomechanics, increased inflammatory burden, or technical procedural difficulty [14]. One possible explanation for these findings is that the analgesic mechanism of RFA-thermal interruption of nociceptive signaling-may be relatively independent of patient demographic characteristics once appropriate anatomical targeting and lesioning are achieved. Consequently, procedural precision and accurate localization of the target nerve may represent more meaningful determinants of treatment durability than baseline patient demographics alone.

​Likewise, no statistically significant differences in repeat RFA frequency were observed between racial groups or between sexes. These findings may suggest that RFA remains an effective interventional option across diverse patient populations without substantial variation in procedural utilization based solely on demographic characteristics. Given the increasing emphasis on equity within pain medicine and rehabilitation, these findings may have important implications for patient counseling and procedural decision-making. Clinicians may reassure patients that demographic factors alone are unlikely to predict the need for repeat RFA procedures or reduced procedural durability.

​The findings of this study should also be interpreted within the broader context of chronic pain as a multifactorial biopsychosocial condition. While demographic variables were not associated with repeat RFA frequency in this cohort, other factors not evaluated in this study may contribute substantially to treatment response and procedural durability. Potential contributors include psychosocial variables such as depression, anxiety, pain catastrophizing, coping strategies, social support, and patient expectations, as well as technical procedural variables including lesion size, target accuracy, needle positioning, and anatomical variability. Previous literature has demonstrated that psychosocial distress and psychiatric comorbidities can influence pain severity, functional outcomes, and treatment perception, potentially contributing to variability in long-term pain management outcomes [20-23]. Future prospective investigations incorporating these variables may provide a more comprehensive understanding of predictors of RFA success and recurrence.

​Several limitations of this study should be acknowledged. First, this study was conducted at a single academic center, which may limit the generalizability of the findings to other patient populations and clinical practice settings. Second, the retrospective design introduced the possibility of residual confounding from unmeasured clinical factors, such as baseline pain severity, disease severity, prior surgical history, prior response to treatment, and concurrent conservative treatments, as well as selection bias related to the exclusion of patients without documented follow-up. Socioeconomic factors such as insurance status, housing stability, and access to care were also not captured in this study. Third, procedural factors were not evaluated and may have influenced outcomes. Variables such as needle gauge, electrode positioning during the ablative process, number of needle passes, ablation temperature, and confirmation of adequate sensory and motor stimulation before ablation may affect lesion size or procedural outcomes. For example, larger-diameter needles can produce larger lesions [8]. Technique may also vary among physicians, with some performing a single 90-second ablation, whereas others may use a different duration or create an additional lesion. These factors were not recorded during the study period. Additionally, although the study was adequately powered overall, certain racial subgroups were underrepresented, potentially limiting the ability to detect smaller subgroup differences. Finally, patient-reported outcomes such as changes in pain severity, functional status, quality of life, and changes in analgesic use, including opioid dosage quantified in morphine milligram equivalents, were not assessed, limiting conclusions regarding the overall clinical efficacy of repeat RFA procedures.

Despite these limitations, this study contributes to the growing body of literature evaluating predictors of RFA efficacy and durability in chronic pain management. Future prospective multicenter studies incorporating psychosocial variables, procedural characteristics, disease severity measures, and patient-reported outcomes are warranted to further clarify the factors associated with long-term RFA success and treatment durability.

## Conclusions

In conclusion, this study demonstrated that demographic variables, including race, sex, age, and BMI, were not significantly associated with repeat radiofrequency ablation (RFA) utilization in patients undergoing chronic pain management. These findings suggest that baseline demographic characteristics alone may have limited utility in predicting procedural durability or the likelihood of requiring multiple RFA interventions. Rather, treatment response may be more strongly influenced by procedural precision, anatomical targeting, disease-specific factors, and psychosocial determinants of pain. As chronic pain remains a complex and multifactorial condition, individualized treatment strategies remain essential to optimizing patient outcomes. Further prospective, multicenter investigations incorporating procedural variables, psychosocial measures, and patient-reported outcomes are needed to better characterize predictors of long-term RFA efficacy and to guide evidence-based patient selection and treatment planning.

## References

[1] Treede RD, Rief W, Barke A (2019). Chronic pain as a symptom or a disease: the IASP classification of chronic pain for the International Classification of Diseases (ICD-11). Pain.

[2] Rikard SM, Strahan AE, Schmit KM, Guy GP Jr (2023). Chronic pain among adults - United States, 2019-2021. MMWR Morb Mortal Wkly Rep.

[3] Zis P, Daskalaki A, Bountouni I, Sykioti P, Varrassi G, Paladini A (2017). Depression and chronic pain in the elderly: links and management challenges. Clin Interv Aging.

[4] Nicholas M, Vlaeyen JW, Rief W (2019). The IASP classification of chronic pain for ICD-11: chronic primary pain. Pain.

[5] Volkow ND, McLellan AT (2016). Opioid abuse in chronic pain--misconceptions and mitigation strategies. N Engl J Med.

[6] Provenzano DA, Holt B, Danko M (2025). Assessment of real-world, prospective outcomes in patients treated with lumbar radiofrequency ablation for chronic pain (RAPID). Interv Pain Med.

[7] Rodriguez-Merchan EC, Delgado-Martinez AD, De Andres-Ares J (2023). Radiofrequency ablation for the management of pain of spinal origin in orthopedics. Arch Bone Jt Surg.

[8] Wray JK, Dixon B, Przkora R (2026). Radiofrequency ablation. StatPearls [Internet].

[9] Chen AF, Khalouf F, Zora K (2020). Cooled radiofrequency ablation compared with a single injection of hyaluronic acid for chronic knee pain: a multicenter, randomized clinical trial demonstrating greater efficacy and equivalent safety for cooled radiofrequency ablation. J Bone Joint Surg Am.

[10] Park D, Chang MC (2022). The mechanism of action of pulsed radiofrequency in reducing pain: a narrative review. J Yeungnam Med Sci.

[11] Sam J, Catapano M, Sahni S, Ma F, Abd-Elsayed A, Visnjevac O (2021). Pulsed radiofrequency in interventional pain management: cellular and molecular mechanisms of action - an update and review. Pain Physician.

[12] Cohen SP, Bhaskar A, Bhatia A (2020). Consensus practice guidelines on interventions for lumbar facet joint pain from a multispecialty, international working group. Reg Anesth Pain Med.

[13] McCormick ZL, Korn M, Reddy R (2017). Cooled radiofrequency ablation of the genicular nerves for chronic pain due to knee osteoarthritis: six-month outcomes. Pain Med.

[14] Searcy S, Mora J, Meroney M (2023). Retrospective review of the efficacy of lumbar radiofrequency ablation for lumbar facet arthropathy: the influence of gender and obesity. Pain Physician.

[15] Rękas-Dudziak A, Brzeziński K, Kotlińska-Hasiec E, Dąbrowski W, Matuła P, Płotek W (2024). A retrospective assessment of the effectiveness of pulsed radiofrequency ablation in the treatment of chronic pain caused by advanced knee osteoarthritis. Anaesthesiol Intensive Ther.

[16] Boody BS, Sperry BP, Harper K, Macadaeg K, McCormick ZL (2022). The relationship between patient demographic and clinical characteristics and successful treatment outcomes after basivertebral nerve radiofrequency ablation: a pooled cohort study of three prospective clinical trials. Pain Med.

[17] Yadav A, Hagedorn JM, D'Souza RS, Engle AM, Deer TR (2021). Effect of patient characteristics on reported outcomes over 12 months following lumbar radiofrequency ablation: a retrospective review of 500 patients. Pain Pract.

[18] Ma Y, Chen YS, Liu B, Sima L (2024). Ultrasound-guided radiofrequency ablation for chronic osteoarthritis knee pain in the elderly: a randomized controlled trial. Pain Physician.

[19] Kose SG, Kose HC, Celikel F, Akkaya OT (2022). Predictive factors associated with successful response to utrasound guided genicular radiofrequency ablation. Korean J Pain.

[20] de Heer EW, Gerrits MM, Beekman AT (2014). The association of depression and anxiety with pain: a study from NESDA. PLoS One.

[21] Harte N, Aaron RV, Bhattiprolu K (2024). The association between anxiety and depression symptoms and pain and function in adolescents and young adults with chronic pain: a meta-analysis. J Psychosom Res.

[22] Khan JS, Hah JM, Mackey SC (2019). Effects of smoking on patients with chronic pain: a propensity-weighted analysis on the Collaborative Health Outcomes Information Registry. Pain.

[23] Karm MH, Kwon HJ, Kim CS, Kim DH, Shin JW, Choi SS (2024). Cooled radiofrequency ablation of genicular nerves for knee osteoarthritis. Korean J Pain.

